# Molecular Docking Studies of HIV-1 Resistance to Reverse Transcriptase Inhibitors: Mini-Review

**DOI:** 10.3390/molecules23051233

**Published:** 2018-05-21

**Authors:** Olga Tarasova, Vladimir Poroikov, Alexander Veselovsky

**Affiliations:** Institute of Biomedical Chemistry, 10 Building 8, Pogodinskaya st., Moscow 119121, Russia; vladimir.poroikov@ibmc.msk.ru (V.P.); veselov@ibmh.msk.su (A.V.)

**Keywords:** HIV-1, reverse transcriptase, resistance, molecular docking

## Abstract

Currently, millions of people are living with human immunodeficiency virus type 1 (HIV-1), which causes acquired immunodeficiency syndrome. However, the spread of the HIV-1 resistance to antiviral agents is the major problem in the antiretroviral therapy and medical management of HIV-infected patients. HIV-1 reverse transcriptase (RT) is one of the key viral targets for HIV-1 inhibition. Therefore, the studies on the combatting the HIV resistance that occurs due to the structural changes in RT, are in great demand. This work aims to provide an overview of the state-of-the-art molecular docking approaches applied to the studies of the HIV-1 resistance, associated with RT structure changes. We have reviewed recent studies using molecular docking with mutant forms of RT. The work discusses the modifications of molecular docking, which have been developed to find the novel molecules active against resistance mutants of RT and/or recombinant strains of HIV-1. The perspectives of the existing algorithms of molecular docking to the studies on molecular mechanisms of resistance and selection of the correct binding poses for the reverse transcriptase inhibitors are discussed.

## 1. Introduction

Reverse transcriptase (RT) of human immunodeficiency virus type 1 (HIV-1) is an important target for design of new anti-HIV drugs. HIV-1 RT inhibitors are used both for treatment of HIV infection and for prevention of mother-to-child transmission of HIV-1 [1]. Small molecules that target the viral proteins responsible for reverse transcription and viral DNA integration are of great importance for pre-exposure HIV-1 prophylaxis [2,3,4,5,6,7]. The appearance of HIV-1 resistance, associated with amino acids substitutions in RT, requires the development of novel approaches that explain the relationships between a particular conformation of HIV-1 RT and drug resistance. These data can be applied for further development of new HIV-1 RT inhibitors and/or prediction of the HIV-1 resistance to antiretroviral drugs [8].

Molecular modeling allows explaining the molecular mechanisms of resistance associated with a particular three-dimensional complex of RT and a small molecule. This method may also be used for revealing new compounds that are active against the mutant forms of HIV-1 RT [9]. Docking-based approaches are useful for predicting the importance of mutations for the resistance of HIV-1 if a data set of the known drugs is employed to perform the molecular docking procedure [10]. Molecular docking can be carried out if data are available on, at least, one complex of protein-ligand (protein-low molecular weight compound) in contrast to the structure-property relationship analysis, which requires the data on a set of low molecular weight compounds and their impact on a particular protein. The molecular docking approaches are repeatedly reviewed [9,11,12,13]. However, recent molecular docking approaches aimed at studying the interaction of small molecules with the resistant RT HIV-1 variants, are not widely discussed. In our mini-review, we focus on the use of molecular docking for the mutant forms of HIV-1 RT. The selection criteria were as follows: (1) the publication describes the application of molecular docking for the development of new compounds that are against RT of resistant HIV strains; (2) new mechanisms of interaction between RT and small molecule were determined using molecular docking or (3) new potentially active RT inhibitors were discovered using molecular docking. In this work, we discuss the causes of HIV-1 RT-associated resistance at the molecular level and data sources for molecular docking approaches.

## 2. Results and Discussion

### 2.1. Molecular Mechanisms of the HIV-1 Drug Resistance to Reverse Transcriptase Inhibitors

#### 2.1.1. Three-Dimensional Structure of HIV-1 Reverse Transcriptase

HIV-1 reverse transcriptase is a heterodimer consisting of two subunits: p66 (560 amino acids, aa) and p51 (440 aa) [14,15]. It has been proposed that p51 subunit is formed from p66 subunit via cleavage by the viral protease [14,15]. The process of RT structural maturation and dimerization has been studied [15,16]. It has been shown that dimerization of RT subunits is a prerequisite for the activity of the enzyme [17,18]. Hence, RT structural maturation is considered as an attractive target for therapeutic intervention [15,16,17].

The functions of p51 were studied earlier [18,19]. Although the direct role of p51 in HIV-1 replication and overall viral production has not been discovered yet, mutational studies provide some hypotheses supporting the influence of p51 on the correct positioning of the RNA/DNA substrate in the Ribonuclease H (RNase H) active site of RT and, thus, on RT activity.

Polymerization starts with the interaction of reverse transcriptase with tRNA primer and RNA/DNA substrate, which in turn leads to the changes in the p66 conformation. To perform polymerization RT needs to bind primer (human tRNA) and template (virus DNA/RNA). Unbounded deoxynucleotide triphosphate (dNTP) molecules are also necessary to build DNA chains.

P66 subunit contains two active sites responsible for (1) interaction with the nucleoside triphosphates (dNTP) resulting in the single-strain DNA synthesis (polymerase activity) and (2) the RNA-DNA hybrid cleavage (ribonuclease, RNase H activity). It is known that interaction of the polymerase domain with both a template and a primer is necessary to perform DNA polymerization. The polymerase domain contains four subdomains shaped like a human right hand: thumb (positions 237–318), fingers (1–85 and 118–155), palm (86–117 and 156–236) and connection (319–426). P51 subunit contains the same subdomains as p66 does, however, the spatial orientation of the domains in p51 differs from those in p66. The binding site of p66 with RNA/DNA formed with fingers, palm and connection of polymerase domain and the whole ribonuclease domain.

The other highly conserved regions of polymerase domain include (1) (YXDD) motif [20] that includes tyrosine (Y183) residue, a variable amino acid X (X184) residue, and aspartic acids 185 and 186 (D185 and D186) residues [20]; (2) K65 and R72 residues [21,22,23]; (3) Y115 residue [24] and (4) Q151 [25]. The mechanisms of interaction of these motifs and residues with the dNTP molecules and the nucleic acid substrate (host tRNA and viral DNA/RNA) during DNA polymerization are reviewed in [25,26].

#### 2.1.2. Mechanisms of Reverse Transcriptase Inhibition

**Figure 1:**
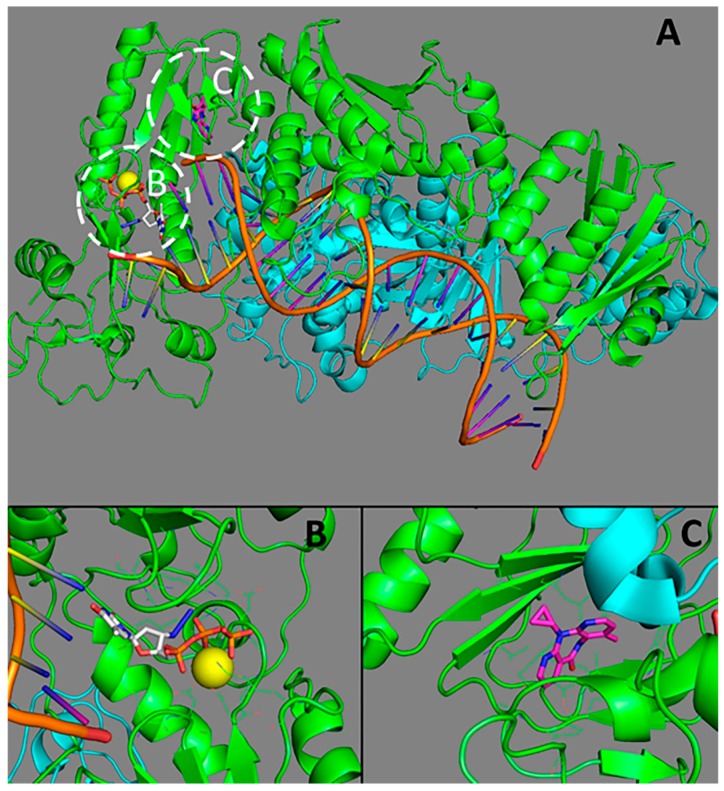
The structure of HIV-1 reverse transcriptase (RT). (**A**) an overall representation of RT; (**B**) the active site of RT (binding site of nucleoside reverse transcriptase inhibitors (NRTI)); (**C**) the allosteric site (binding site of non-nucleoside reverse transcriptase inhibitor (NNRTI)). p66 subunit is represented in green, p51 subunit is given cyan; Mg^2+^ is in yellow; zidovidine is white; nevirapine is magenta. The structures from the Protein Data Bank [27] PDB ID 3V4I [28] and 4PUO [29] were used.

There are two main types of RT inhibitors. The first ones, nucleoside reverse transcriptase inhibitors (NRTIs) are the structural analogs of the nucleosides used by RT to build DNA chain and interact with RT in its active site (Figure 1). Clinically approved NRTIs include didanosine, lamivudine, abacavir, stavudine, tenofovir disoproxil. Unlike the real nucleosides, inhibitors lack 3′-OH group that leads to the chain growth termination and stops the DNA synthesis.

Any set of HIV-1 mutations can be classified according to the main mechanism leading to reverse transcription disruption. For example, several studies describe two distinct possible mechanisms of RT-NRTI interaction disruption: exclusion and excision [30].

There are several mutations and its combinations leading to the resistance to NRTIs (see Table 1). Some of them cause resistance due to the recognition of the inhibitor and disruption of the interaction, or by the so-called exclusion mechanism. The mutation M184V/I is located in palm subdomain and results in the blockade of emtricitabine (FTC) and lamivudine (3TC) interaction due to steric hindrance with beta-branched amino acids [31]. The resistance to didanosine and tenofovir occurs due to mutations L74V and K65R. There are studies, which partially explain the mechanisms of resistance associated with L74V and K65R mutations. There are several combinations that include Q151M, M184V/I and K65R/E and lead to the resistance to most of the nucleoside inhibitors [31,32,33,34]. The role of these mutations is considered in the studies of Iyidogan and Anderson [30] and Garforth et al. [23]. It is supposed, that K65 interacts with gamma-phosphate of the incoming dNTP and, probably, K65R mutation disrupts that interactions [35]. There is the hypothesis that the K65R replacement may influence on the conformations of K65 and R72 resulting in the resistance to tenofovir [36]. We should emphasize that although some suggestions about molecular mechanisms of resistance have been proposed, the mechanism of resistance for K65R RT has not been determined yet. Therefore, it is still difficult to use this information for molecular docking approaches because there are many objects (including RT, DNA, RNA, dNTPs) which interact with each other during reverse transcription. Some details about intermolecular interactions between RT and an inhibitor are still not well understood.

Other mutations that can potentially cause resistance may be classified as the thymidine analog mutations. They include the following ones: M41L, D67N, K70R, L210W, T215F/Y, K219E/Q [37]. The precise impact of each mutation on the resistance mechanism to NRTIs is not well known. In addition, there are some suggestions about a role of mutations in the region around position 69, located in the fingers subdomain (D67N, K70, etc.).

Earlier, we have mentioned the general set of mutations associated with the resistance to at least one antiretroviral drug. Some of them may be prevalent for a particular nucleoside/non-nucleoside RT inhibitor. Each drug can be characterized by its own pattern of mutations leading to HIV-1 resistance to that drug (Table 1, see also [38]). For instance, mutations that lead to a single amino acid substitution at position 65 (K65) are often involved in resistance to most NRTI drugs including abacavir (ABC), emtricitabine (FTC), lamivudine (3TC), stavudine (d4T) and, in particular, tenofovir (TDF) [39]. It is noteworthy to say that for some reverse transcriptase inhibitors it is possible to assume the most likely mechanism of stability (i.e., exclusion, excision), while for some others the most probable mechanism is still unknown.

Some relatively rare and new mutations (for example, Q151L) have been described previously [40]. The authors suggest that this mutation causes the impairment of the incorporation of active form of NRTI (for instance, GS-9148-diphosphate) [40].

There are many attempts to classify the mutations leading to HIV resistance to NRTIs according to their molecular mechanisms, which might be very complicated and involve multiple pathways. Also, novel and under-investigated mutations may occur resulting in new potential mechanisms of resistance.

Non-nucleoside inhibitors (NNRTIs) bind with reverse transcriptase in the allosteric site located about 10 Å away from the active site of RT at the subunits interface biochemical and X-ray data enabled to reveal that NNRTIs are non-competitive inhibitors of RT [41]. Clinically approved NNRTIs include nevirapine, efavirenz, etravirine, rilpivirine. The hydrophobic pocket of allosteric site contains the amino acid residues: L100, K101, K103, V106, T107, V108, V179, Y181, Y188, V189, G190, F227, W229, L234, Y318 of p66 and E138 of p51 [42,43].

Mutations causing resistance to NNRTIs are around the binding pocket of the NNRTIs and contain the following single replacements K103N, Y181C, L100I, K101E, V106A, V179D, Y188L, G190A, P236L (see Table 1). Combinations of these mutations lead to a significant decrease in susceptibility to the inhibitor. Several particular mechanisms related to resistance to NNRTIs are described in publications [37,43,44,45].

In particular, the most frequent mutations often resulting in HIV-1 drug resistance are regularly considered in the molecular docking studies: K103N, Y181C, Y188L/C/H/F, L100I. 

A probable mechanism leading to the resistance to RT in position Y181C and Y188L/C/H/F is associated with the loss of *π*-*π* stacking between the changed amino acids residues and small molecules [44]. The loss of antiretroviral activity in K103N mutants can be associated with several different mechanisms. In particular, it has been proposed that K103N mutant RT can have a set of hydrogen bonds, while wild-type RT does not have such bonds (for instance, hydrogen bonds may be formed between N103 and Y188). The formation of hydrogen bond between the amino acid residues may have an impact on the inhibitor accessibility to reach the binding site for NNRTIs. Also, it is proposed that replacement of lysine (K) with asparagine (N) might lead to changes in hydrophobic and electrostatic interactions, which are supposed to play an important role in the binding site of NNRTIs [46]. The L100I mutation can be associated with the loss of van der Waals contacts between Y181 and Y188 changing the geometry of a possible binding site of NNRTIs [46]. The mutations in β12–β13 (i.e., mutations in positions 227–235) may influence both polymerase and ribonuclease activity of HIV-1 RT [47].

There are several mutations located in the connection subdomain and in RNA H active site, which increase resistance to both NRTIs and NNRTIs [48]. For instance, the mutations that can confer resistance are: G333D, A360V, N348I. Typically, these mutations are common for HIV variants, extracted from samples of patients who have undergone treatment. It has been shown that there are no contacts between nucleic acid substrate and the amino acid residues, located in RNA H active site. There are the data that supports the hypotheses about antagonistic effects of A360V and some thymidine-analog mutations and synergistic effect of G333D and M184V mutations. The relationship between viral fitness and resistance level, which is caused by these mutations is under investigation [48].

Some relatively new mutations and their effect on HIV-1 RT-associated resistance to both NRTIs and NNRTIs are described [49,50]. The authors supposed that minor changes in the p66 thumb subdomain can result in the re-positioning of the nucleic acid or inhibitor in the NRTI binding site. The changed conformation of the binding site may provide a distinction between a nucleic acid and inhibitor. However, the authors mentioned that this mechanism needs to be described in precise detail. Thus, we may propose that the data on the rare mutations are not enough to use them in search of new promising inhibitors using molecular modeling. However, another point of view is that a possible mechanism of resistance due to rare mutations may be partially explained using this method.

Molecular docking requires three-dimensional (3D) structure of RT with the amino acid changes occurring due to mutations. The data on the molecular mechanisms of resistance to NRTIs and NNRTIs can be obtained either from various biochemical methods or based on the data on three-dimensional complexes of inhibitors with RT obtained using X-ray techniques. Knowledge about the three-dimensional structures of enzyme-inhibitor complexes is especially important for the molecular modeling.

### 2.2. Molecular Docking in Studies of HIV-1 Drug Resistance to Reverse Transcriptase Inhibitors

Molecular docking is one of the broadly used computational procedures in the computer-aided drug design [9,12,51,52]. Typically, a set of ligands is positioned within the protein’s binding site (for more details of this method, see [51,52,53,54,55]). For each pose of a ligand in the binding site, the interaction energy is estimated using the scoring function. Then, the best poses of this ligand, according to the calculated scores can be determined either manually or automatically. 

To obtain the three-dimensional structure of mutant RT, the general molecular modeling software may be used. The 3D structure of mutant RT optimization can be done by energy minimization or, preferably, by simulation of molecular dynamics using AMBER (University of California, San Francisco, CA, USA, http://ambermd.org/), GROMACS (GROMACS University of Groningen, Netherlands, http://www.gromacs.org/) or other packages [55]. Other methods and applications of molecular docking in the field of HIV reverse transcriptase inhibition are reviewed partially in [9,12,13]. There are some approaches directed to the estimation of a binding energy minimum through the generation of several possible conformations of ligand in the protein active site. Typically, these approaches require the results of molecular docking. Thus, they represent post-docking optimization or post-docking protocols [56,57,58,59,60,61,62,63]. It is supposed that such an optimization of docking results can help to improve the results of molecular docking with a flexible ligand/proteins binding site and distinguish the compounds that can bind with a protein target from false positives [59].

#### 2.2.1. Data on Three-Dimensional Complexes

The Protein Data Bank (PDB) [27] is the main source for the three-dimensional structures of the proteins and protein-ligand complexes. By 20 April 2018, PDB contained over 250 three-dimensional complexes of HIV-1 reverse transcriptase found with over 90% identity with the amino acid sequence of gag-pol protein representing wild-type HIV-1 reverse transcriptase sequence (part of gag-pol sequence retrieved from [64]). We have filtered the relevant structures using the queries to PDB through the web-interface. Then, relevant structures were manually inspected. As of 20 April 2018, we have identified more than 190 ligand-protein complexes that can be potentially used in molecular docking studies. The PDB identifications (IDs) of complexes with the most common mutations, associated with HIV resistance are listed in Table 2. The complexes selected contain proteins of both wild-type and resistant HIV-1 variants.

#### 2.2.2. Applications of Molecular Docking for the Development of New HIV Reverse Transcriptase Inhibitors

There are a lot of studies on HIV-1 RT inhibitors where molecular docking is used for one purpose or another. Most studies are directed to the molecular docking of potential inhibitor with the wild type RT. In our review we consider the approaches where molecular docking was performed with mutant forms of RT. Some recent approaches, providing new insights on the mechanism of the HIV-1 resistance to both NRTIs and NNRTIs are presented in Table 3 and discussed below.

Usually, molecular docking is used in the HIV-1 resistance studies for the two main purposes. First, using this procedure, the authors try to explain the interaction of the experimentally tested inhibitors with a protein. Currently, such studies are widely used in tandem with experimental findings and seem to be important in the understanding of the ligand-protein interactions. Second, molecular docking is used in virtual screening to find new reverse transcriptase inhibitors. The latter often requires the combination with other computational and/or experimental methods. Here, we discuss both applications of molecular docking.

In a publication by Fourati et al. the influence of the rare K65E replacement on the resistance of HIV-1 to NRTIs is discussed [65]. The relationship between the K65E amino acid replacement and HIV-1 resistance to inhibitors was studied using an experiment that assessed the cytotoxicity of MT-2 cells infected with HIV-1. The experimental studies of the K65E influence on the RT-NRTI binding were not productive because of a significant impairment of viral fitness of clones with K65E replacement. Molecular docking was used to illustrate a possible role of K65E in HIV-1 resistance to NRTIs. The PDB complex of RT-DNA (1RTD) was used for docking. The mutations were generated computationally using MacroModel 9.2 software (Schrödinger, LLC, New York, NY, USA). The authors supposed that the binding of the inhibitor with RT is not possible if the distance between the 3′ OH group of nascent DNA chain and certain atoms of inhibitor is over 6 Å by the docking results. The authors showed that the distance between the tenofovir and the nascent DNA chain was increased (d = 10.3 Å) in the presence of K65R. The researchers made a suggestion that K65E mutation may induce resistance to NRTIs by shifting the binding site of inhibitors that result in decreasing free energy of binding. It is noteworthy to say that the molecular docking results enrich the data on a probable molecular mechanism of resistance associated with the changes of K65, where K65 replacement influenced the interactions of K65 with incoming dNTPs [35]. We suppose further molecular modeling studies can shed the light on the several new pathways leading to K65-associated resistance.

An impact of A98S polymorphism on the response to the combinations of tenofovir (TDF), and emtricitabine (FTC) was studied earlier [66]. First, the statistical analysis has been performed using multivariate logistic regression to study the impact of A98S on the therapeutic outcome. Then in vitro studies were conducted using peripheral blood mononuclear cells (PBMC). The viral activity was measured as a p24 production using Elisa. Docking was used to explain the experimental results at the level of intermolecular interactions. In particular, the molecular docking results supported the hypotheses about an increase in TDF and FTC binding affinity for RT. The binding energy for TDF-mutant A98S RT was slightly higher (−73.047 kcal/mol) than that of TDF-wild-type of RT (−62.074 kcal/mol). The authors supposed that TDF can form a hydrogen bond with the phosphate of deoxyguanosine at position 22 in mutant RT, while this contact is missing in the wild-type (WT) and M184V best configurations. Similarly, FTC-RT interaction revealed an increased number of contacts between FTC and palm domain of RT. For example, a slight increase in the binding affinity between FTC and RT (101.392 kcal/mol for mutant RT vs. 92.140 kcal/mol for wild-type RT) may be a result of conformational changes occurring due to A98S mutations. The authors also supposed that the complex RT double-mutant A98S + M184V-FTC has a slightly more stable configuration than in RT M184V-FTC (−79.756 kcal/mol vs. M184V −74.312 kcal/mol). These results are in agreement with a higher number of contacts between RT and the inhibitor. In particular, that might be a result of conformational changes occurring due to A98S mutations and leading to the shift of small molecule to YMDD motif (a variant of an YXDD motif, which contains M184 residue). Thus, molecular docking studies support the positive relationships between the occurrence of A98S mutation and viral RNA in blood plasma, revealed in multivariate regression models. This study is an example of the application of molecular docking procedure to investigate the effect of minor RT mutation onto enzyme activity.

Most studies were devoted to molecular docking of NNRTIs. Some recent examples of molecular docking of NNRTIs to resistant HIV RT variants are considered further.

A set of diarylpyridine derivatives (DAPYs) were synthesized and tested against wild-type RT and five RT mutant variants [67]. Twenty-one compound showed moderate inhibitory activity against wild-type variant of RT., and three compounds were active against both wild-type and K103N RT variants. Molecular docking of the most active compound in wild-type and K103N mutant variants of RT was used to clarify the possible binding mode. According to the results obtained, the compound under investigation was located within the hydrophobic sub-pocket near the residues Tyr181, Tyr188, Phe227, Trp229, Leu234, Pro236, Tyr318, interacting with them. These results partially support the hypotheses about the importance of hydrophobic contacts for allosteric binding of NNRTIs. Also, the results of docking enabled to suppose that the studied compound interacts with Glu138 and Lys101 at the entrance of NNRTI binding pocket and, thus, prevents the loss of activity in mutant K103N variants.

The diarylpyrimidine analogs were tested and the mechanism of their activity was studied using molecular docking [68] In particular, the authors examined the mechanism of the binding between the compounds and the double mutant of HIV-1 RT (V106A and F227L) with one of the synthesized compounds. The loss of contacts between the chemical compounds and the mutated V106A amino acid residue were found. Also, the loss of interaction between aromatic groups of chemical compound and F227L mutated amino acid residue was observed. The authors supposed that the loss of contacts could decrease binding affinity between the small molecule and double V106A and F227L mutant, which was shown experimentally in their work.

Several experiments were performed to identify the binding mode of a new (+)-calanoide analog, F18, with RT, indicating that the binding modes of F18 and nevirapine (NVP) can be different [69]. Using molecular docking a set of most favorable complexes of F18-RT and NVP-RT were obtained.

Then, the clusterization of binding poses allowed to reveal the similar stable conformations of F18 and NVP in NNRTIs (both, wild-type and L100I variants of RT). But the orientation of the F18 in Y181C variant of RT is completely different from that of wild-type and L100I variants. The molecular docking results along with the experimental studies demonstrated several differences in the binding mode of F18 with p51 of RT and NVP with p51, respectively.

In the study of Samanta et al. several molecular docking programs were considered, including Autodock (versions 4.2, Vina; The Scripps Research Institute, La Jolla, CA, USA; http://autodock.scripps.edu/) and ArgusLab (developed by M. Thompson, version 4.0.1). These programs were applied to investigate the binding modes between catechol diether based NNRTIs and RT wild-type, as well as Y181C and Y181C/K103N mutants [70]. As an extension of the molecular docking programs applied, quantum mechanics and molecular mechanics calculations (ONIOM2) calculations have been performed to estimate the binding energies. Based on the results of molecular docking, it has been demonstrated that the *π*-*π* stacking interactions between Tyr181 and the small molecules are lost in both the mutated variants RT (Y181C) and RT (K103N/Y181C). These results are partially in agreement with earlier suggestions about the mechanisms of resistance for Y181C and K103N mutants.

A set of dihydropyrimidinone (DHPM) derivatives, which probably act as NNRTIs and their activity against K103N/Y181C mutant RT are described [71]. A set of marine diterpenes (dolabelladienotriol, THD and its derivatives) has been docked with various mutant forms of RT containing mutations: K103N, V106M, Y188L; V106M, P225H; V106A, Y181I; V106A, Y181C, G190S; K103N, Y188L; K103N, Y188L, G190E [72]. Autodock 4 was used. The authors performed a thorough analysis of molecular modeling results and revealed that THD forms some contacts with mutant RT, which are absent in the wild-type RT. For instance, Van der Waals interactions were observed with Lys102, Val189, Gly190 and Phe227 in a mutant form of RT containing V106A, Y181I mutations; Phe227 in a mutant form containing V106A, Y181C, G190S. THD can form hydrogen bonds with Ala190 in a mutant RT with K103N, G190A and K103N, Y181C, G190A and some others. The experimental studies were performed prior to the docking procedure, so the results obtained partially explained the molecular mechanism of marine diterpenes as the previously discovered micromolar RT inhibitors.

The mechanisms of action of rilpivirine were investigated using molecular modeling methods [73]. The authors used molecular docking with K103N mutant. Molecular dynamic simulations were performed using AMBER (version 12., University of California, San Francisco, CA, USA). The binding energy of mutant RT in the complex with an inhibitor was then calculated. Thus, the trajectories of bound and unbound RT were collected and the influence of rilpivirine on the three-dimensional structure changes of RT was evaluated. The analysis of the results obtained made it possible to suggest that linker nitrogen atom of rilpivirine can form a hydrogen bond with Lys101 in the mutant RT, while N atom can form a hydrogen bond with Lys103 in the wild-type enzyme. Undoubtedly these results support the new hypotheses for the potent NNRTI discovery of highly active NNRTI using both protein-based and ligand-based approaches.

There are several approaches leading to the selection of small compounds that are active against HIV RT using molecular docking. However, docking is not as widely used for this purpose as for the explanation of molecular mechanisms. Also, molecular docking is often employed for wild-type RT rather than for mutant RT variants.

It may be concluded that the existing application of the molecular docking to HIV-1 RT resistance studies lead mainly to the discovery of new molecules targeted at well-investigated mutant RT forms [67,68,69,70,71,72]. But some approaches are directed to research studies of known molecules targeted at comparatively rare RT mutants [65,66,73]. The considered applications of molecular docking demonstrated good results when the effect of a particular mutation on the level of resistance needs to be discovered or explained. Generally, molecular docking procedure required a thorough analysis of the data by experts, and the automated molecular docking is not widely used.

### 2.3. Perspectives of Molecular Docking in the Studies of HIV-1 Resistance

Summarizing the results of the studies on the application of molecular docking to the development of new HIV RT inhibitors we can make several conclusions. Molecular docking is used for designing NRTIs and NNRTIs. However, the number of studies dedicated to the NNRTIs design is significantly higher. This fact can be explained by the difficulties to properly estimate the binding energy between NRTIs and RT, due to the mechanism of NRTIs action related to competitive binding.

While several possible mechanisms for HIV-1 resistance to NRTIs have been studied, described, and classified, there are still many controversial hypotheses about the mechanisms of the resistance, and a role of each mutation in the level of resistance is not known with high confidence [74]. Thus, the possibilities of using molecular docking in the studies of HIV-1 resistance to NRTIs are rather limited. Probably, the newly discovered mechanisms of HIV-1 resistance to NRTIs may lead to the possibilities of molecular docking application for the development of highly active NRTIs. 

Molecular docking studies with mutant RT forms require the data about the possible binding site(s) and certain binding poses of the compounds. Such investigations are restricted if there are no crystallographic complexes of mutant resistant variants of RT with known inhibitors. Such cases are typical for the recently discovered mutations.

While virtual screening of compounds that are active against mutant RT forms is not widely used, an application of molecular docking to virtual screening of compounds, active against wild-type of RT can be performed [75,76,77,78,79,80,81]. Typically such studies provide some insights into intermolecular interactions affecting both wild-type and mutant enzymes.

There is a study [81], where a comparison of several molecular docking approaches to a potential NNRTIs selection was provided. In particular, the authors compared Glide, FlexX, Molegro Virtual Docker, AutoDock Vina, and Hyde on their ability to predict RT inhibitory activity of 111 1,2,4-triazole and 76 azole derivatives. They showed that the correlations between the binding energy estimated and the experimental values of half maximal effective concentration (EC_50_) were highly dependent on the ligand set. Nevertheless, these findings support the hypothesis about the general possibility of the application of docking procedure for selection of the compounds that can be potentially active on HIV RT. 

In the study of Vadivelan et al., several computational methods including docking were used to screen a library of 10,000 structures of chemical compounds [82]. Molecular docking was used at the latest stage of screening to investigate the binding affinity for 77 compounds filtered at the previous stages. The favorable conformations were analyzed to select the most potent compounds for further testing. This study does not contain any data on the experimental testing using resistant variants of HIV RT. However, this study is interesting for further analysis as it contains a set of computational methods combined with a virtual screening procedure. Also, retrospective validation using known HIV RT inhibitors and prospective validation using newly synthesized compounds were performed.

In particular, in the study of Tintori et al. [83], the molecular docking Gold software [84] was used to perform virtual screening of a chemical library of tetrahydropyrimido[2,1-f]purinedione analogs, which are potential inhibitors of RT p51–p66 dimerization. The potential binding site corresponded to a tryptophan-rich region of p66, suggesting that it is involved in p51–p66 interaction during dimerization. The structures of compounds were arranged according to the docking scores and were further manually inspected. The most important residues involved in p51–p66 interaction were supposed W402 and W410. The structures of compounds potentially interacting with these residues were selected for further in vitro testing. In vitro studies confirmed the results of docking procedure. Although the investigation of small molecules with mutant strains of RT has not been provided, the authors supposed that the newly obtained p51–p66 inhibitors can be used for the inhibition of any mutant strains of RT, probably because p66 contains conservative region (W398–W402, W406, W410, W414) and the mutations in that region do not occur.

Therefore, the applications of the molecular docking algorithms to automated selection of the compounds active against the mutant variants of RT are highly in-demand. As have already been mentioned, the number of ligand and protein complexes is rather high, if a set of ligands should be docked into a set of proteins, where each protein contains a set of mutations. Therefore, the systematic application of molecular docking for virtual screening of RT inhibitors requires a significant amount of computing resources and thus is related to big data approaches and their application in the field of cheminformatics. We suppose that these new approaches, libraries or application programming interfaces directed to the molecular docking with the enzyme with multiple mutations and their combinations may be one of the possible ways of evolution for molecular docking approaches.

While detailed enumeration of all mutation combinations requires the development of new software, libraries, and technical support, there is a need for new approaches aimed at automatic selection of favorable conformations.

The development of approaches, which might improve prediction of the RT inhibition using molecular docking [85,86,87,88,89] is another future direction of the molecular docking approaches. We suppose that creation of new methods, which provide the possibility of taking into account small molecules pharmacophores together with docking results might be helpful.

New approaches aiming at the improvement of the predictive value of the molecular docking with mutant forms of RT are also highly in-demand. We propose that the development of various machine learning methods for improvement of docking scoring functions may lead to significant enhancement of the molecular docking predictive value. In particular, there is a number of studies where various machine learning methods were applied for prediction of binding affinity of various ligand sets [90,91,92,93]. For example, molecular docking scores may be used as one of the descriptors employed for machine learning prediction [91]. We propose that it may be widely used in HIV-1 resistance studies because there are data on mutations and resistance associated with them. The data on the relationships between the occurrence of a particular mutation and reduced susceptibility to a drug may be used as the descriptors in machine learning together with docking results. We suggest that it can lead to significant improvements in the prediction of drug activity due to the possibility of including the data on the three-dimensional complex RT-inhibitor in the model.

## 3. Conclusions

In this mini-review, we have considered recent approaches directed to the molecular docking of both nucleoside and non-nucleoside inhibitors to the resistant variants of RT, published in the last decade. We also discussed common mutations leading to resistance to NRTIs and NNRTIs, which are typically used in computer-aided drug design. Application of molecular docking, as well as the limitations of this approach, were considered. Molecular docking is a powerful and useful procedure for understanding the binding mode of newly synthesized and tested compounds.

## Figures and Tables

**Table 1:** The mutations and their combinations leading to drug resistance.

Drug Name	Class	The Major Positions and Mutations Leading to Resistance
Lamivudine	NRTI ^1^	K65R, M184V/I
Emtricitabine	NRTI	K65R, M184V/I
Abacavir	NRTI	K65R, K70I, L74V/I, Y115F, M184V/I
Tenofovir	NRTI	M41L, K65R, K70R, T215F/Y
Zidovudine	NRTI	M41L, D67N, K70R, L210W, T215F/Y, K219Q/E
Nevirapine	NNRTI ^2^	L100I, K101E/P, K103N/S, V106A/M, Y181C/I/V, Y188L/C/H, G190A/S/E/Q, M230L
Efavirenz	NNRTI	K103N/S, V106A/M, Y181C/I/V, Y188L/C/H, G190A/S/E/Q, M230L
Etravirine	NNRTI	L100I, K101E/P, Y181C/I/V, Y188L/C/H, G190A/S/E/Q, M230L
Rilpivirine	NNRTI	L100I, K101E/P, Y181C/I/V, Y188L/C/H, G190A/S/E/Q, M230L

^1^ NRTI: nucleoside reverse transcriptase inhibitor; ^2^ NNRTI: non-nucleoside reverse transcriptase inhibitor.

**Table 2:** Complexes of both wild-type and mutant HIV-1 reverse transcriptase with various inhibitors in Brookhaven Protein Data Bank (PDB) identification (IDs).

Mutation/Wild-Type Protein	Number of Complexes	PDB IDs
Wild-type	120	1C0T; 1C0U; 1DLO; 1DTQ; 1DTT; 1FK9; 1HMV; 1HNI; 1HNV; 1HVU; 1HYS; 1IKW; 1JLQ; 1KLM; 1N5Y; 1N6Q, 1R0A; 1REV; 1RT1; 1RT2; 1RT4; 1RT5; 1RT6; 1RT7; 1RTD; 1RTH; 1RTI; 1RTJ; 1S6P; 1S6Q; 1S9E; 1S9G; 1SUQ; 1T03; 1T05; 1TKT; 1TKX; 1TKZ; 1TL1; 1TL3; 1TV6; 1TVR; 1VRT; 1VRU; 2B5J; 2B6A; 2BAN; 2BE2; 2HMI; 2HND; 2I5J; 2JLE; 2OPP; 2RF2; 2RKI; 2VG5; 2VG6; 2VG7; 2WON; 2YKM; 2YKN; 2YNG; 2YNH; 2YNI; 2ZD1; 3C6T; 3C6U; 3DI6; 3DLE; 3DLG; 3DRP; 3DYA; 3E01; 3FFI; 3HVT; 3I0R; 3I0S; 3IG1; 3IRX; 3IS9; 3ISN; 3ITH; 3KJV; 3KK1; 3KK2; 3KK3; 3KLF; 3LAK; 3LAL; 3LAM; 3LAN; 3LP0; 3LP1; 3LP2; 3M8P; 3M8Q; 3MEC; 3MEE; 3NBP; 3QIP; 3T19; 3V4I; 3V6D; 3V81; 4B3O; 4B3P; 4B3Q; 4I7F; 4ID5; 4KV8; 4LSL; 4NCG; 4PQU; 4PUO; 4PWD; 4Q0B; 4R5P; 5CYM; 5HLF; 1BQM
Y188C	4	1JLE; 1JLG; 2OPS; 2YNF
K103N	18	1FKO; 1FKP; 1HPZ; 1HQE; 1HQU; 1IKV; 1IKX; 1IKY; 1SV5; 2WOM; 3DOK; 3DM2; 3DRS; 3MED; 3MEG; 3T1A; 3TAM; 1SV5
K103N/Y181C	7	2IAJ; 2IC3; 3BGR; 3DM2; 4RW4; 4RW7; 5FDL
L100I/K103N	1	2ZE2
Y181C	9	1C1B; 1C1C; 1JKH; 1JLA; 1JLB; 1JLC; 1UWB; 3DRR; 4RW6
Y188C	4	1JLE; 1JLF; 1JLG; 2OPS
Y188L	2	1BQN; 2YNF
Other mutations or combinations	53	1EET; 1EP4; 1J5O; 1LW0; 1LW2; 1LWC; 1LWE; 1LWF; 1QE1; 1RT3; 1S1T; 1S1U; 1S1V; 1S1W; 1S1X; 2HNY; 2HNZ; 2OPQ; 2OPR; 2OPS; 2ZE2; 3DMJ; 3DOL; 3JSM; 3JYT; 3KLE; 3KLG; 3KLH; 3KLI; 3MED; 3QLH; 3QO9; 4DG1; 4G1Q; 4H4M; 4H4O; 4I2P; 4I2Q; 4ICL; 4IDK; 4IFV; 4IFY; 4IG0; 4IG3; 4KFB; 4KKO; 4KO0; 4KSE; 4LS; 4MFB; 4O44; 4O4G; 4RW8; 4RW9; 4WE1; 4ZHR; 5C24; 5C25; 5C42; 5CYQ; 5HBM

**Table 3:** Some examples of molecular docking applied to study HIV-1 resistance to NRTIs and NNRTIs.

Mutati-Ons	PDB ID	Mutant RT Preparation	Class of Drug or Chemical	Docking Algorithm, Package	Energy Minimization Algorithm in Post-Docking Protocol	Reference
K65E	1RTD	Computationally MacroModel 2.0	NRTIs, marketed drugs	Autodock 4.0	AMBER (ligand)	[65]
A98S		Computationally	NRTIs, marketed drugs	-	N/D *	[66]
WT K103N	3MED	PDB complexes	NNRTIs diaryl-pyridine derivatives (DAPYs)	Surflex-Dock (Sybyl-X 2.0)	N/D	[67]
V106A and F227L	2ZD1	Computationally	NNRTIs diaryl-pyrimidine analogs	Sybyl-X 1.2	N/D	[68]
WT L100I Y181C	1VRT 1S1U 1JLB	Experimental (PDB)	NNRTIs (+)-calanoide analog	Autodock 4.2	AMBER (ligand-protein energy calculation) Conformation clusterization using RMSD	[69]
WT Y181C	4H4M 4RW6 4RW4 4RW8 4RW9 4RW7	Computationally using PDB complexes 4RW6, 4RW4, 4H4M	NNRTIs catechol diether based NNRTIs	Autodock 4.2Autodock Vina ArgusLab 4.0.1	ONIOM2 calculations	[70]
WT K103N Y181C	3BGR 4I2Q 4I2P	PDB complexes	NNRTIs dihydro-pyrimidinone (DHPM) derivatives, probably NNRTIs	Molegro Virtual Docking	N/D	[71]
A set of various mutations	1HNV	Computationally	NNRTI marine diterpenes	Autodock 4.2	N/D	[72]
K103N	3MED	PDB complex	NNRTI rilpivirine	AutodockRaccoon	Molecular dynamics using AMBER 12 (F99SB) force field	[73]

* N/D—not determined.
